# Universal Internucleotide Statistics in Full Genomes: A Footprint of the DNA Structure and Packaging?

**DOI:** 10.1371/journal.pone.0112534

**Published:** 2014-12-01

**Authors:** Mikhail I. Bogachev, Airat R. Kayumov, Armin Bunde

**Affiliations:** 1 Radio Systems Department & Biomedical Engineering Research Center, Saint Petersburg Electrotechnical University, Saint Petersburg, Russia; 2 Department of Genetics & Institute of Fundamental Medicine and Biology, Kazan (Volga Region) Federal University, Kazan, Tatarstan, Russia; 3 Institut für Theoretische Physik, Justus-Liebig-Universität Giessen, Giessen, Hessen, Germany; National Institute of Genomic Medicine, Mexico

## Abstract

Uncovering the fundamental laws that govern the complex DNA structural organization remains challenging and is largely based upon reconstructions from the primary nucleotide sequences. Here we investigate the distributions of the internucleotide intervals and their persistence properties in complete genomes of various organisms from *Archaea* and *Bacteria* to *H. Sapiens* aiming to reveal the manifestation of the universal DNA architecture. We find that in *all* considered organisms the internucleotide interval distributions exhibit the same 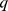-exponential form. While in prokaryotes a single 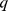-exponential function makes the best fit, in eukaryotes the PDF contains additionally a second 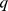-exponential, which in the human genome makes a perfect approximation over nearly 10 decades. We suggest that this functional form is a footprint of the heterogeneous DNA structure, where the first 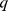-exponential reflects the universal helical pitch that appears both in pro- and eukaryotic DNA, while the second 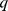-exponential is a specific marker of the large-scale eukaryotic DNA organization.

## Introduction

Understanding the complex structure of DNA as the carrier of genetic information is one of the major challenges of modern science. The architectural complexity of eukaryotic DNA is associated with its extensive functional versatility including accomodation and realization of genetic information as well as controlling the cell activity and its adaptation to various conditions. It is known that the human genome is about three orders of magnitude larger than the typical microbial genome, while its coding part is only about 1.5 orders of magnitude larger. This indicates that the increase of the genome size during evolution is mainly caused by the accumulation of the noncoding DNA. While the functionality of the noncoding DNA is still under debate, there is a major agreement about its architectural role in the formation of the complex eukaryotic DNA structure. This suggests that the genomic evolution is not limited to the introduction of new genes but also includes considerable complication of the DNA spatial structure. Understanding the DNA structural evolution is especially important since it significantly contributes to the control of the gene expression, DNA replication, recombination and repair mechanisms [1], [2].

The DNA consists of two complementary polynucleotide chains which at small scales form a double helix with a helical pitch of about 10–11 base pairs (bp) [1], [2] that is universal for all cellular life. At larger scales, the DNA structure varies considerably between different domains of life, the simple *prokaryotes* exemplified by *Archaea* and *Bacteria* and the *eukaryotes* characterized by a cell that contains a nucleus (a large group of organisms ranging from yeast, fungi and plants to animals including *H. Sapiens*). While in *Bacteria* the DNA is located in a relatively free manner in the cytoplasm, with random attachments to the cell membrane and without any characteristic structural scales, in *Archaea* the DNA is additionally wrapped around the histones. In contrast, in the *eukaryotes* the DNA structure is more complex and, additionally to these two structural levels, constitutes several other packaging levels with larger characteristic scales. For an extensive review on the eukaryotic DNA structure, we refer to [2].

The primary structure of DNA is determined by a sequence that consists of four nucleotides, namely adenosine (A), cytosine (C), guanosine (G) and thymidine (T). The second polynucleotide chain can be normally reconstructed from the first one due to their complementarity, provided that A is opposed to T and G is opposed to C, and thus statistical analysis can be performed on a single sequence. The two types of base pairs have considerably different bonding energies characterized by the bond enthalpies −11.8 for A:T and −23.8 kcal/mol for G:C, respectively [3]. Nevertheless the occurrence of either G:C or A:T in the primary sequence leads to the identical tertiary architecture of the base pair and thus their alteration does not perturb the double helix structure [1].

It has been revealed earlier that the DNA sequences exhibit long-range correlations (LRC) with rather monofractal properties [4]–[9]. It has been established that there are two scaling regimes in the DNA sequences that are separated by a prolonged crossover typically between 100 bp and 1 kbp. Below the crossover the correlations are characterized by Hurst exponents 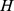 close to 0.5 for the prokaryotes and close to 0.6 for the eukaryotes. Above the crossover, 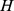 is around 0.8 in both domains. Grossberg *et al.* first proposed that the long-range correlations in the DNA primary sequences are related to its three-dimensional structure [10]. Next the LRC have been associated the formation of the DNA bending profile [11], and the two separate scaling regimes were attribued to the different hierarchical levels of the DNA structure [12], [13]. The relationship between the LRC in the DNA primary sequence and its elastic bend stiffness related to the DNA loops formation has been further investigated [14], [15].

The original approach to the LRC in DNA is based on the “DNA walk”, which increases by one when a pyrimidine (C or T) is observed and decreases by one when a purine (A or G) is observed in a DNA sequence [4]. Here we follow a more direct approach to the DNA primary structure based on the persistence properties of the intervals between the same nucleotides (A-A, C-C, G-G, T-T) in the DNA sequence. The central quantities here are the distribution of the intervals and their autocorrelation function that are signatures of both linear and nonlinear correlations in the analysed DNA sequences. In random sequences, the probability density function (PDF) of the intervals is a simple exponential 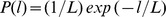, where 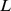 is the average interval length, and the intervals are uncorrelated. In LRC sequences similar nucleotides tend to follow each other, and thus short (long) intervals are more likely to be followed by short (long) intervals. When there are purely linear correlations in the sequence, one expects that the PDF follows a stretched exponential 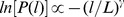, with exponent 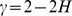. The intervals are also LRC and their autocorrelation function (ACF) decays by a power law 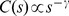 with the same correlation exponent 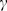 [16]–[18]. In the presence of nonlinear correlations, the PDF gets even broader and decays by a power-law 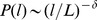, where the exponent 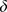 changes with the strength of the LRC in the data [19], [20]. In some complex systems with nonlinear LRC like returns in financial markets exceeding a certain threshold also 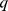-exponential PDFs of the intervals have been observed [21]. In both cases, the ACF of the intervals decays by a power law [16], [18], [19].

## Materials and Methods

**Figure 1:**
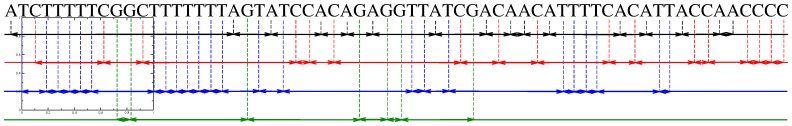
The procedure of the assessment of the four internucleotide interval sequences from the DNA primary sequence.

We assessed the complete genomes of organisms at different evolutionary positions ranging from *Archaea* (48 species) and *Bacteria* (72 species) to various eukaryotes including *H. Sapiens* from the NCBI GenBank [22]. From each genomic sequence we obtained the series of consecutive intervals between the same nucleotides (A-A, C-C, G-G, T-T). The procedure of the assessment of the four internucleotide interval sequences from the DNA primary sequence is shown in Fig. 1. We focused on the two major quantities characterizing the intervals series, the probability distribution functions (PDFs) and the autocorrelation functions (ACFs).

To obtain the distribution, we first counted the number of occurrences of intervals of a certain size of 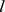 nucleotides, which constituted the histrogram 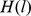. To obtain the probability density function (PDF), we next divided 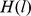 by the total number of fragments in the genome. By definition, the PDF is normalized, 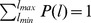, where 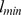 and 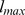 are the shortest and the longest intervals observed in the studied sequence. Since large fragments occur very rarely, the statistics becomes poor for large 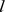, and the functional form of the PDF can no longer be observed visually.

In order to improve the statistics gradually with increasing size 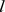, we have chosen logarithmic binning, which is widely used in statistical physics in order to determine the behavior of the tails of the distribution. In the logarithmic binning, one counts the number of fragments with sizes between 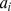 and 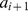, 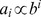, where 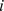 is the number of bin. We have tuned the parameter 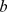 to achieve the best visualization of our results, in particular 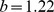 for the PDFs obtained from the single DNA sequences and 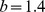 for the total PDFs over several DNA sequences. The respective 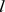 value was associated with the center of the bin in log-scale, i.e., at the geometric average. For example, when the bin included values from 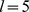 to 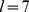, we averaged 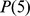, 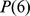 and 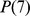, with the associated 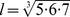, respectively. Once a bin which contained less than three occurrences was found, the analysis was stopped. For obtaining the functional form of 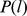 over several decades, we typically used a double-logarithmic presentation.

Finally, in order to eliminate the changes in the average size of fragments, and to concentrate solely on the shape of the distributions, we used the average interval 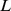 as a characteristic scale for the size distributions. We rescaled the PDFs by dividing the sizes 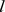 by their mean value 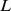 for every particular genome. To keep the normalization, we also multiplied the PDFs by 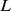, and thus finally obtained 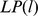 as a function of 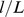.

Next we calculated the linear two-point autocorrelation function (ACF) 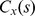 of the interval series 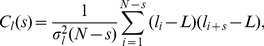
(1)where 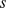 is the scale parameter and 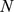 is the total number of intervals in the sequence, and applied a similar logarithmic binning procedure to it.

## Results

### Internucleotide interval distributions

**Figure 2:**
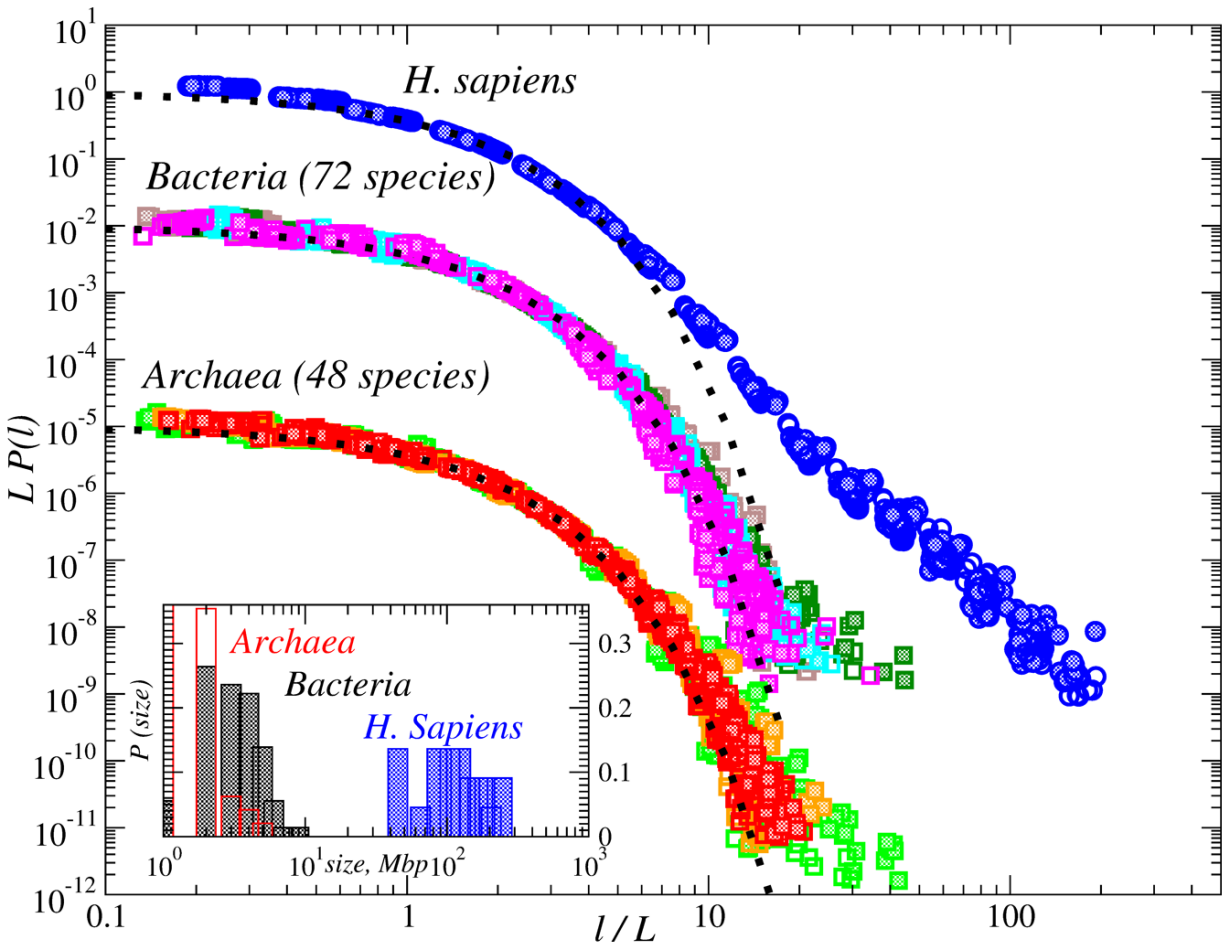
PDFs of the inter-nucleotide intervals A-A, T-T (open symbols); G-G, C-C (full symbols) in the DNA sequences from *Archaea* (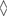) (48 species), *Bacteria* (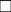) (72 species) and *H. Sapiens* (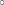) (22 chromosomes). For comparison, dotted lines show corresponding exponential PDFs. The inset shows the size distribution plots of the DNA sequences considered.

Figure 2 shows the PDFs of the inter-nucleotide intervals in the DNA of *Archaea* (48 species), the believed predecessor of all other forms of life, *Bacteria* (72 species) and *H. Sapiens* (22 chromosomes) calculated from the complete genomes obtained from the NCBI GenBank [22]. The PDFs are provided in scaled form, i.e., the intervals 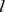 are given in units of the average interval 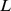 (around 4), and the PDFs are multiplied by the same 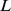 value to keep the normalization. For comparison, the figure shows also simple exponential 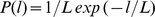 distributions (by dotted lines. At scales 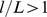 the empirical PDFs are significantly broader than the simple exponential distributions rejecting the hypothesis of the random positioning of nucleotides. In archaeal and bacterial genomes, the simple exponential appears close to the lower bound for the empirical PDFs, while the upper bound resembles a power law at the tail of the distribution. Additionally pronounced scattering at the tail of the PDFs can be observed and thus a particular functional form can hardly be determined from the analysis of individual sequences. This scattering could be attributed both to the heterogeneity of the considered *Archaea* and *Bacteria* as well as to the finite size effects taking into account the limited size of the considered DNA sequences (see the inset in Fig. 2). In contrast, in the *H. Sapiens* genome the scattering is much less pronounced, and the empirical PDFs exhibit a specific two-compound shape with clearly significant deviations from a single exponential distribution.

**Figure 3:**
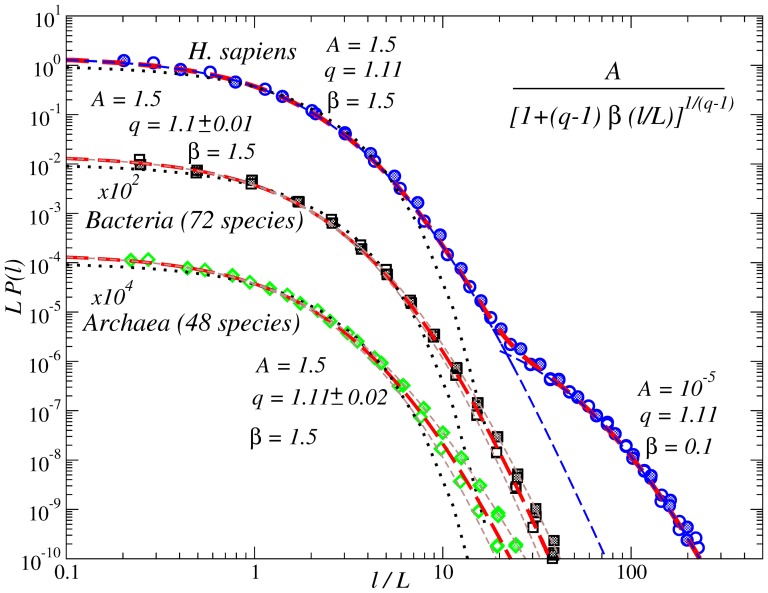
PDFs of the inter-nucleotide intervals A-A, T-T (open symbols); G-G, C-C (full symbols) in the DNA sequences from *H. Sapiens* and *Bacteria* full genomes (in scaled form). Dashed lines show the best fits by a 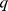-exponential distribution 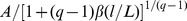. While in *Bacteria* the approximation by a single 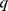-exponential with 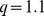 and 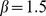 is possible, in *H. Sapiens* a sum of two 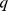-exponentials with 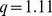 and 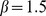 and 0.1 makes the best fit. To avoid overlapping, the PDFs for *Bacteria* are shifted downwards by two decades. For comparison, dotted lines show corresponding exponential PDFs.

In order to determine the PDFs more accurately, instead of considering the individual histograms 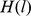 and PDFs 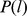, we next calculate the total histograms 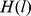 of inter-nucleotide intervals over *all Archaea* and *Bacteria* genomes as well as the total histogram over all 22 chromosomes in the *H. Sapiens* genome. The results are shown in Fig. 3 in the units of the respective average intervals 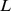. The figure shows that the total PDF in *Bacteria* can be well approximated by a single 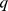-exponential distribution 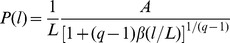
(2)with 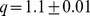, 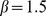 and 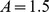 over 8 orders of magnitude limited by the genome size. The 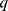-exponential distribution is a special case of the generalized Pareto distribution 
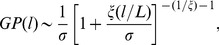
(3)where 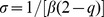 and 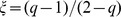. The 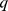-exponential distribution extremizes, under simple constraints, the nonadditive entropy which is the generalization of the Boltzmann-Gibbs entropy [26]. In the limit 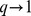 the 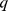-exponential distribution reduces to a simple exponential. At small arguments 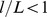 the 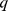-exponential it behaves as 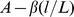 for all 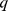 values. A similar functional form but with 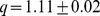 can be observed in the total PDF for the *Archaea*. It is also notable that in prokatyores the PDFs for the stronger bonded nucleotides are slightly broader than for the weakly bonded ones.

In the human genome, the description by a single 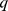-exponential is valid only at small and intermediate scales 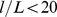, while at large scales 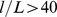 another 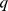-exponential *with the same* 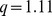 as in *Archaea*, but now with 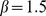 and 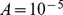 makes a perfect fit. If we add both 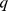-exponentials, we obtain an excellent fit over nearly 10 orders of magnitude limited by the human chromosome size (see the size distribution plot in the inset of Fig. 3). It is remarkable that in the *H. Sapiens* genome there is a perfect collapse for the internucleotide interval distributions for each of the nucleotides A, C, G or T.

Given 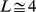 the first 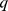-exponential is valid until approximately 100 bp that is close to the characteristic scale of the DNA wrapping cycle (146 bp in *H. Sapiens*) [2], [27] and thus might correspond to the first two DNA structural levels, namely the double helix and the DNA wrapping around the histones (nucleosome formation) [2], [12], [13]. The second 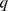-exponential likely reflects the eukaryotic DNA organization at larger scales. It is interesting that, despite of the apparent otherness of different DNA structural levels, the 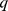 value is identical, that suggests striking similarities in their optimization principles.

**Figure 4:**
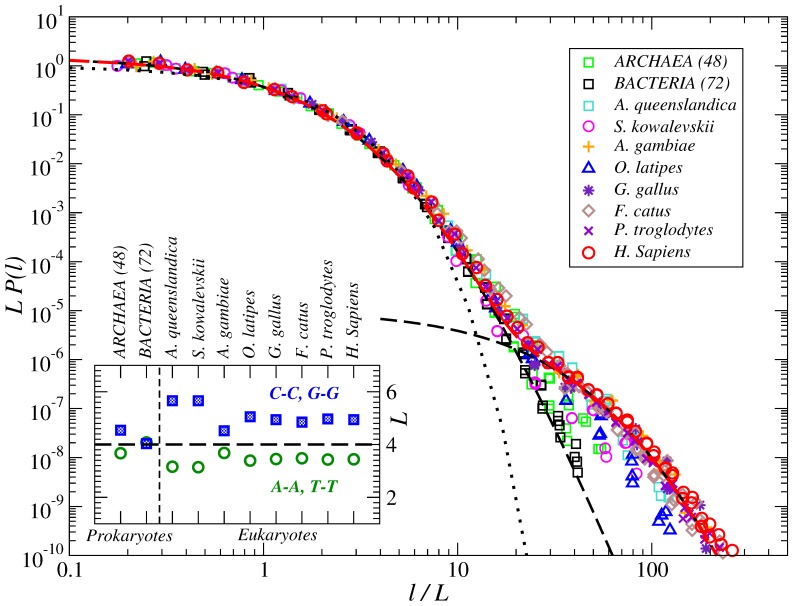
PDFs of the internucleotide intervals in the DNA from full genomes of ten different organisms at different evolutionary positions from *Archaea* and *Bacteria* to *H. Sapiens*. The thin dashed line shows an approximation by a single 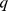-exponential, while the thick dashed line shows an approximation by a sum of two 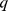-exponentials. For comparison, the dotted line shows the corresponding exponential PDF. The inset shows the evolution of the average interval 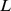 separately for strongly (G:C) and weakly (A:T) bonded nucleotides.

Figure 4 shows (in addition to the results of Fig. 3) the scaled PDFs of internucleotide intervals for seven more eukaryotic species at various evolutionary levels such as *A. queenslandica* (sponge native to the Great Barrier Reef), *S. kowalevskii* (marine acorn worm), *A. gambiae* (malaria mosquitoes), *O. latipes* (Japanese rice fish), *G. gallus* (chicken), *F. catus* (domestic cat) and *P. troglodytes* (common chimpanzee). The functional fits in the figure are identical to those in Fig. 3. The inset shows the average intervals 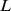 for different nucleotides. The figure shows that, despite of the discrepancy in the average interval 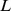 (shown in the inset of Fig. 4), after rescaling the PDFs in *all* higher eukaryotes obey a similar functional form as in *H. Sapiens*. We like to emphasize that for all 10 studied examples the first part of the distribution up to about 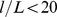 is identical supporting the hypothesis that the observed 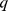-exponential is a footprint of the universal DNA double helix structure that is independent of the evolutionary position of the organism.

Figure 4 additionally indicates that some deviations in the large-scale part of the distribution can be observed for several organisms at intermediate evolutionary positions such as *A. queenslandica*, *S. kowalevskii* and *O. latipes*. The figure shows that the PDFs still follow the universal 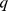-exponential for 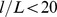. At larger scales there are moderate deviations from the second 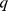-exponential (universal for higher eukaryotes). Besides their evolutionary positions, we like to note that these organisms are all water living with environmentally dependent body temperature and thus the deviations from the universal PDF could be a reflection of their adaptation to the living environment associated with more pronounced thermodynamical constraints.

The inset in Fig. 4 shows that the same water living organisms are characterized by the largest average interval for G and C, i.e., their genomes has the lowest fraction of G:C base pairs and the highest fraction of A:T base pairs among considered species, respectively. Due to the differences in their bonding energies, the relative fractions of G:C and A:T base pairs (GC-/AT-content) are closely associated with the optimal environmental temperature that is limited by the DNA thermostability at the high end and by the energy required for the DNA unwinding during replication and the low end. On the other hand, broader interval distributions for larger average intervals is a typical sign of multifractality [18], [19]. However, in multifractal data this effect is commonly observed over a wide scale range, contrast to the observations in Fig. 4. It is also known that in eukaryotic genomes GC-rich and AT-rich regions exhibit alterations that are associated with the DNA replication domains and thus the average G:C or A:T intervals over the whole genome are often not representative for most of its particular fragmetns.

**Figure 5:**
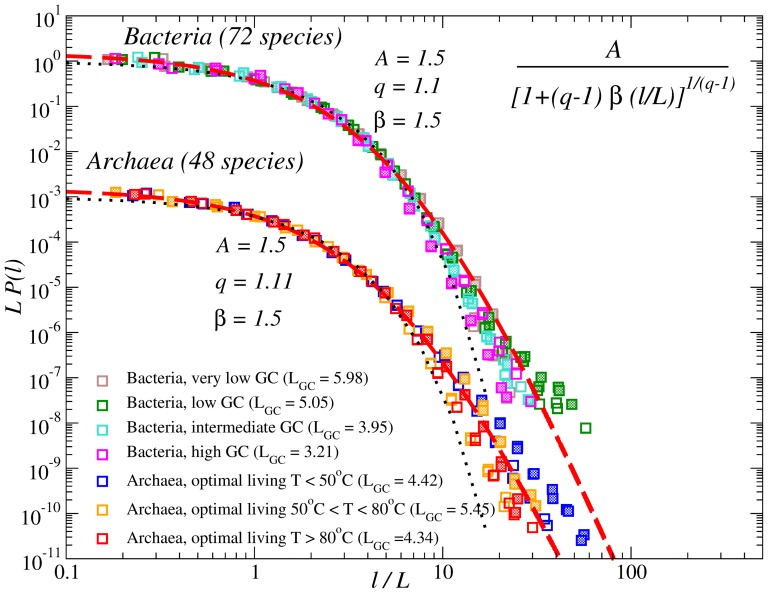
PDFs of the internucleotide intervals in the DNA from full genomes of *Bacteria* classified into four groups according to the fraction of G and C in their genomes (GC-content) and *Archaea* classified into three groups according to their optimal living temperature, from normal environment to extermophiles. The dashed lines show approximations by a single 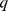-exponential with 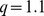 for *Bacteria* and 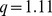 for *Archaea*. Open symbols denote distributions for A and T, while full symbols denote distributions for G and C.

To further evaluate the effects of the G:C/A:T content and of the environmental temperature, we considered prokaryotes with less complex genome structure. Figure 5 shows the PDFs of the inter-nucleotide intervals in the DNA from full genomes of *Bacteria* classified into four groups according to the fraction of G and C in their genomes and *Archaea* classified into three groups according to their optimal living temperature, from normal environment to extermophiles. The figure shows that in *Bacteria* the distributions for larger average intervals 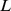 appear slightly broader. Additionally for some groups in both *Bacteria* and *Archaea* the PDFs for G and C appear typically broader than for A and T that could be attributed to more pronounced thermodynamical constraints on the location of the stronger bonded nucleotides. In *Archaea* the dependence of the PDFs on the environmental temperature for different species can be also clearly observed and is especially pronounced when comparing the normal (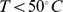) and the extermophile (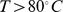) groups, despite of their average intervals between G:C base pairs 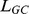 being nearly identical. Our results indicate that moderate deviations from the universal PDFs observed under peculiar conditions like environmentally dependent body temperature in a wide range and/or extreme living temperatures cannot be fully described by the average interval 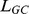 as a single parameter within a framework of a simple multifractal model.

**Figure 6:**
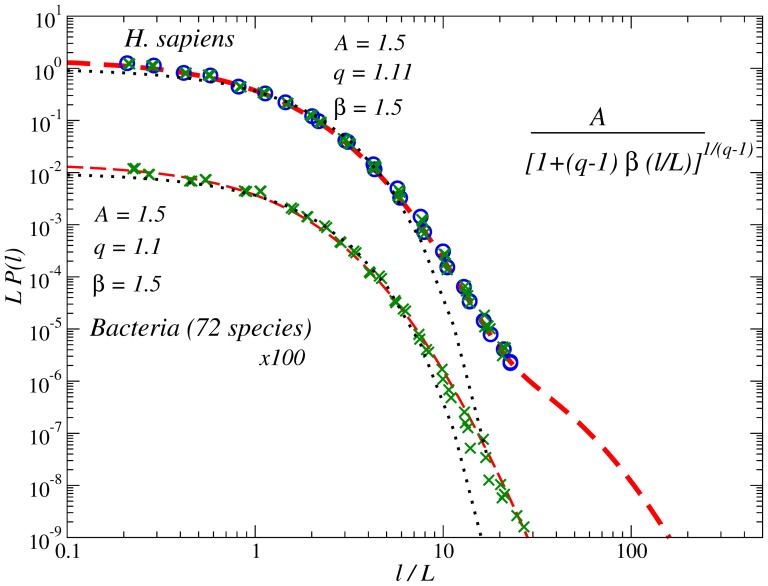
Similar to Fig. 3 but shows the corresponding PDFs for the transcribed DNA (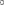) and for the complementary DNA (cDNA, 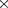). Fits are the same as in Fig. 3. To avoid overlapping, the PDFs for *Bacteria* are shifted downwards by two decades. For comparison, dotted lines show corresponding exponential PDFs.

To check whether the observed functional forms are related to the coding/noncoding DNA fragments, we also considered the PDFs for the transcribed DNA sequences (which contain both the coding exons and the noncoding introns) and the complementary DNA (cDNA) sequences which contain only coding parts of DNA. Figure 6 shows the corresponding results for the same organisms as in Fig. 3. Since the sequences considered here are quite short, we used the semi-logarithmic presentation to best distinguish from exponential functional form. The figure shows explicitly that both in *Bacteria* and in the *H. Sapiens* genomes at least in the small- and medium-scale regime 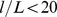 the fit by a single 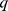-exponential is also valid for the transcribed and for the cDNA sequences. Since there are no introns (noncoding intragenic DNA) in *Bacteria* their transcribed DNA contains solely coding DNA (that corresponds to the eukaryotic cDNA). Both the transcribed and the complementary DNA sequences are relatively short and the large-scale behaviour could not be determined accurately.

**Figure 7:**
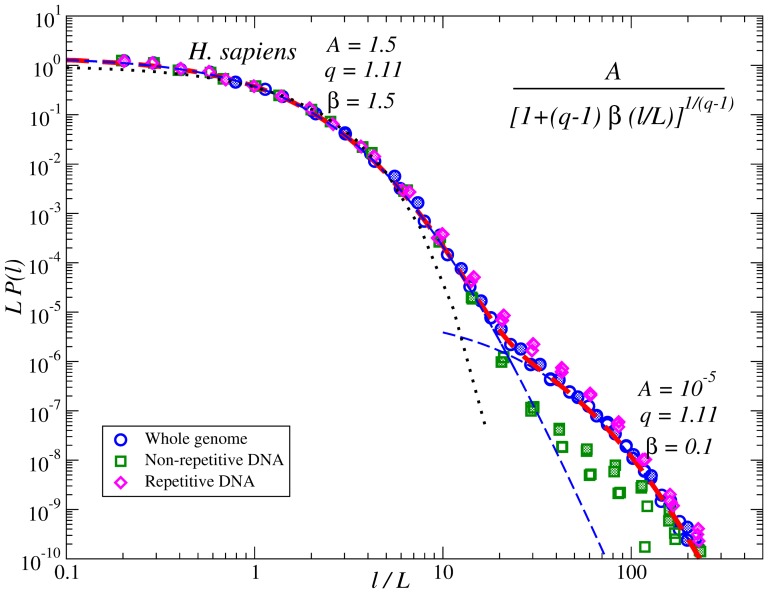
Similar to Fig. 6 but shows the corresponding PDFs for the human genome after elimination of the repeat-masked DNA. For comparison, the dotted line shows corresponding exponential PDF.

In the *H. Sapiens* genome we also considered repetitive DNA that is found in multiple copies throughout the genome. Since the repetitive DNA consists of relatively short fragments, for further analysis we constructed artificial sequences by concatenating repetitive DNA fragments recongnized by the repeat-masker algorithm [23], [24] and the remaining non-repetitive fragments between them, respectively. The PDFs of internucleotide intervals for such sequences are shown in Fig. 7. The figure shows that, while for the repetitive DNA the PDFs follow roughly the same functional form with double 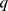-exponential shape, for the remaining non-repetitive DNA significant deviations from this behaviour can be observed. This further indicates that, while the first 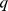-exponential is universal for all DNA fragments, the second 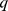-exponential is entirely determined by the noncoding DNA, especially by the intergenic regions where the repetitive DNA is mainly located.

### Internucleotide interval arrangement

**Figure 8:**
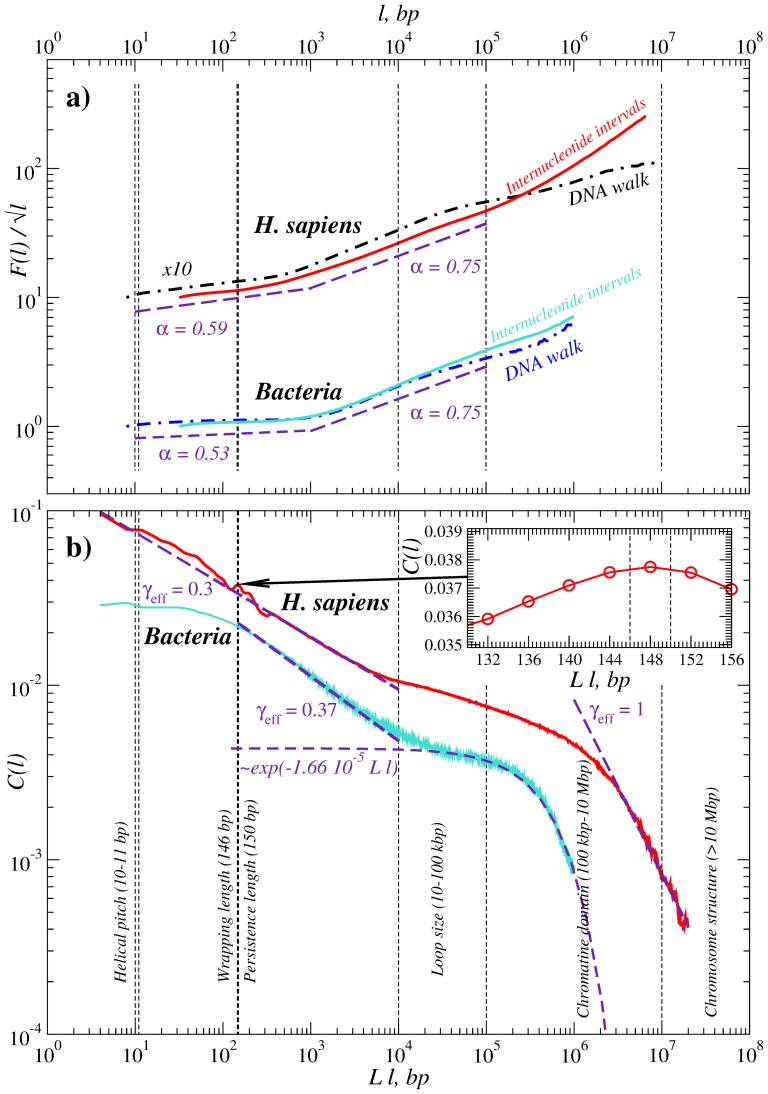
Comparison of the internucleotide interval statistics with the DNA walk analysis: (a) DFA fluctuation functions of the internucleotide intervals (full lines) and of the DNA walks (dashdot lines) and (b) the ACFs of the inter-nucleotide intervals (full lines), all provided for the DNA sequences of *H. Sapiens* (upper curves in each panel) and *Bacteria* (lower curves in each panel) full genomes. For the internucleotide interval sequences, the arguments are multiplied by the average interval 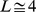 to provide all results in the same units of base pairs. Vertical dashed lines refer to the approximate boundaries of characteristic scaling regimes for different hierarchical levels of eukaryotic DNA packaging structure (exemplified for *H. Sapiens*, following [2]).

To evaluate the inter-nucleotide interval arrangement we also studied the autocorrelation function (ACF) of the interval sequences. For comparison with earlier results based on the the DNA walk analysis, we have calculated the fluctuation functions using second-order detrended fluctuation analysis (DFA) [7] exemplified for the DNA walks of the *H. Sapiens* and *Bacteria* genomes that are shown in Fig. 8(a). Additionally to the DNA walks, we also calculated the DFA for the internucleotide interval sequences, shown in the same figure. To improve the presentation, we have divided the fluctuation functions 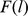 by the square root of their arguments 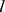 such that the absence of correlations corresponds to the horizontal line in the plot. The figure shows that both fluctuation functions closely reproduce the previous findings of [12], [13]. The empirical exponents of the observational data at small scales are 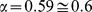 for *H.sapiens* and 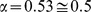 for *Bacteria*, while at large scales 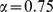 in both pro- and eukaryotes, that is consistent with the typical 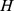 values obtained earlier by wavelet-based analysis in [12], [13]. In our case the crossover appears closer to 1 kbp in both cases, that can be attributed to the higher order detrending used in the DFA analysis (for more details of the effect of the detrending order on the crossover position, we refer to [25]).

Finally, the ACFs for the same internucleotide interval sequences are shown in Fig. 8(b). The figure shows that in the human genome the ACF up to about 10 kbp can be reasonably well approximated by a power-law (PL), 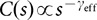, with an effective correlation exponent 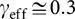, that is consistent with the 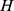 values around 0.8. Our results however indicate that the same correlation exponent 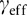 also characterizes the ACF of the intervals at the smaller scales below 200 bp. Notable violations from this behavior could be attributed to the characteristic scales of the DNA packaging structure like the helical pitch at 10–11 bp and the wrapping length at 146 bp that shows up as a spike in the ACF (see the inset in Fig. 8(b)).

In contrast, in *Bacteria* the power law regime can be observed only between 200 bp and 10 kbp with a slightly larger correlation exponent 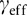 about 0.37, that is consistent with the 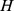 values about 0.8 observed in earlier studies by DNA walk analysis [12], [13]. Below 100 bp the ACF demonstrates a crossover that ends up with a plateau below approx. 40 bp, with a single notable deviation around 10–11 bp corresponding to the helical pitch, that is the only characteristic scale in the prokaryotic DNA packaging.

Despite the considerable discrepancy of the shape of the ACF obtained for the internucleotide intervals in the bacterial and in the human genome, the persistence in the arrangement of nucleotides at scales below 10 kbp both in *Bacteria* and *H. Sapiens* could be interpreted in the framework of the same simple model, where long-range correlations and random “white” noise are superimposed in the interval sequence. It has been shown recently that in the simulated LRC data, the lag 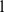 ACF is given by 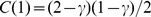 [28], yielding 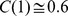 for 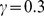 and 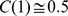 for 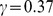. However, the observed 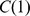 values in Fig. 8 are considerably lower, in particular 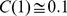 for the human DNA and 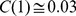 for the bacterial DNA. This indicates that the interval sequences, while exhibiting LRC, also contain additive random “white” noise, which is much more pronounced in bacterial than in the human genome. For an analytical treatment of the superimposed LRC and random noise, we refer to [28].

At large scales above 10 kbp in *Bacteria* the ACF follows a clear exponential decay over the next two decades corresponding to the rather randomized organization of the DNA-membrane attachments to the cell membrane [29]. In contrast, in the *H. Sapiens* genome in the same scale range there is a crossover to a regime with even more pronounced LRC indicating persistence in the large-scale structural organization. This crossover is consistent with the borderline between the chromatin “compaction” and “looping” regimes. The breakdown of LRC in the human DNA occurs well above 1 Mbp that is two orders of magnitude higher than in *Bacteria*. The rapid decay of the ACF with 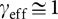 well above 1 Mbp suggests rather uncorrelated arrangements at these scales. For a more detailed overview of the eukaryotic DNA structural levels, we refer to Fig. 1 in [2], p.49.

## Discussion

The observed universality in the functional form of the internucleotide interval distributions indicates the universality of the DNA compaction in the eukaryotic cell nucleus while prokaryotic (bacterial and archaeal) DNA is spreaded over the cell space in a relatively random manner. It has been recently revealed that the DNA structural organization and packaging contributes significantly to its robustness against UV radiation, chemical agents, electromagnetic fields and other external stress factors [30]–[33]. The observed striking universality in the internucleotide interval distributions could be attributed to the similarity in the cell stress pattern perceived by all eukaryotic species from their living environment that leads to stunningly similar structural optimization. The universal 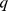-exponential fit as a typical background distribution could also be used when investigating deviations from the typical pattern in eukaryotic DNA as indicators of truncations caused by mutation, insertions of mobile genetic elements and viruses, or specific adaptation to environmental conditions.

In the interval arrangement, the difference in the additive “white” noise level in *Bacteria* and in *H. Sapiens* can explain in addition to the discrepancy between the ACFs of their internucleotide interval sequences, also the differences in the earlier reported wavelet-based fluctuation functions of the respective DNA walks [12], [13]. Since in *Bacteria* the noise is very pronounced, it completely overwhelmes the LRC at small scales resulting in vanishing DNA walk correlations with H close to 0.5 [12], [13]. In contrast, in the *H. Sapiens* genome the noise is less pronounced, and thus the superimposed LRC is not completely hidden at very small scales as in *Bacteria*, but gives rise to an effective Hurst exponent 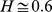. At scales between 100 bp and 1 kbp there is a prolonged crossover to the regime where LRC effects dominate the noise effects and the effective correlation exponent obtained here is consistent with the Hurst exponents obtained earlier by wavelet-based analysis [12], [13]. It is known that in the presence of additive noise fluctuation analysis methods create artificial crossovers like this. Taking into account that the DNA structure at very small scales well below 100 bp is rather similar in pro- and eukaryotes, we suggest that the amount of “white” noise in the arrangement of nucleotides is the only difference, and the respective crossover appears to be an artifact of the analysis technique.

Finally, we like to note that the 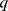-exponential description of distribution functions has been found useful in many other complex systems [21], [34]–[37]. For an extensive review, we refer to [35]. Recently the broad occurrence of Pareto-tailed distributions in a number of economic, social and biological systems has been attributed to similar optimization principles in a common entropy maximization framework [38]. Also very recently 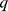-exponentials have been observed in the distributions of interevent times in seismic data [39]. In another recent application in financial markets, also the interoccurrence times between daily losses exceeding a certain threshold have been analyzed [21]. It has been found that the distribution of the interoccurrence times also follows a 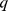-exponential, where the parameter 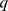 only depends on the average interoccurrence time 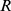 and not on the respective asset (stocks, commodities, or currency exchange rates). We have found that for 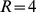, which corresponds to 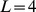 in the DNA example, the same 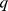-exponential with the same parameters 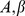 and 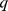 describes the distribution of the interoccurrence times over the first four decades of magnitude. Moreover, the validity of exactly the same approximations has been also confirmed for the financial data with minute time resolution [40]. Due to the limited statistics of the financial data, the behaviour at larger scales cannot be studied. We consider an accidental coincidence as unlikely. The coincidence may indicate that for the DNA structure as well as for the dynamics of the financial markets similar optimization strategies hold.

## Conclusions

In summary, we have investigated the DNA structure using the statistics of internucleotide intervals. The advantage of this approach is that it does not require generation of secondary synthetic sequences like DNA walks. We have shown explicitly that the distribution of the internucleotide intervals exhibits a remarkably universal 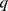-exponential form over nearly five orders of magnitude independently of the evolutionary position of the organism that reflects the universality of the small and intermediate scale DNA structure in all organisms. Differences in the distributions can be observed at large internucleotide intervals, where a second 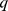-exponential *with the same* 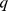 *value* is added for eukaryotes only and thus appears to be a specific marker of the universal large-scale eukaryotic DNA structure. We suggest that this striking universality in the statistical laws governing the DNA primary sequences is a footprint of the DNA tertiary architecture that has undergone similar evolutionary structural optimization due to the similarities in the cell stress pattern perceived by various eukaryotic species from the living environment. We have also shown that the persistence in the arrangement of the intervals is consistent with the hierarchy of the DNA structural organization and reflects the heterogeneity of the optimization patterns at different scales. Moreover, since the direct analysis of the interval sequences is capable of tracking complex models like superposition of LRC and random noise where the interpretation of the DNA walk analysis is complicated a better understanding of the LRC effects in the DNA can be achieved. Another advantage of the interval approach is that it can be easily extended to the analysis of intervals between di- or trinucleotides as well as various combinations of nucleotides.

Finally, we like to emphasize striking similarities between interval distributions in the DNA sequences and in the financial markets, that might indicate similar optimization principles in these very different complex systems.
